# Brain Structure Segmentation from MRI by Geometric Surface Flow

**DOI:** 10.1155/IJBI/2006/86747

**Published:** 2006-01-04

**Authors:** Greg Heckenberg, Yongjian Xi, Ye Duan, Jing Hua

**Affiliations:** ^1^ Department of Computer Science, College of Engineering, University of Missouri-Columbia, Columbia 65211-2060, USA; ^2^ Department of Computer Science, College of Liberal Arts and Sciences, Wayne State University, Detroit 48202, USA

## Abstract

We present a method for semiautomatic segmentation of brain structures such as thalamus from MRI images based on the concept of geometric surface flow. Given an MRI image, the user can interactively initialize a seed model within region of interest. The model will then start to evolve by incorporating both boundary and region information following the principle of variational analysis. The deformation will stop when an equilibrium state is achieved. To overcome the low contrast of the original image data, a nonparametric kernel-based method is applied to simultaneously update the interior probability distribution during the model evolution. Our experiments on both 2D and 3D image data demonstrate that the new method is robust to image noise and inhomogeneity and will not leak from spurious edge gaps.


Hindawi Publishing Corporation
International Journal of Biomedical Imaging
Volume 2006, Article ID 86747, Pages 1–6
DOI 10.1155/IJBI/2006/86747

### Brain Structure Segmentation from MRI by Geometric Surface Flow

**Greg Heckenberg,[1] Yongjian Xi,[1] Ye Duan,[1] and Jing Hua[2]**

[1] *Department of Computer Science, College of Engineering, University of Missouri-Columbia, Columbia, MO 65211-2060, USA*
[2] *Department of Computer Science, College of Liberal Arts and Sciences, Wayne State University, Detroit, MI 48202, USA*

Received 1 August 2005; Revised 22 September 2005; Accepted 28 September 2005

Recommended for Publication by Ming Jiang

We present a method for semiautomatic segmentation of brain structures such as thalamus from MRI images based on the concept of geometric surface flow. Given an MRI image, the user can interactively initialize a seed model within region of interest. The model will then start to evolve by incorporating both boundary and region information following the principle of variational analysis. The deformation will stop when an equilibrium state is achieved. To overcome the low contrast of the original image data, a nonparametric kernel-based method is applied to simultaneously update the interior probability distribution during the model evolution. Our experiments on both 2D and 3D image data demonstrate that the new method is robust to image noise and inhomogeneity and will not leak from spurious edge gaps.

Copyright © 2006 Greg Heckenberg et al. This is an open access article distributed under the Creative Commons Attribution License, which permits unrestricted use, distribution, and reproduction in any medium, provided the original work is properly cited.

### 1. INTRODUCTION

Thalamus is the relay center for nerve impulses in the brain. It mediates communication among sensory, motor, and associative brain regions. Axons from almost every sensory system connect here as the last site before the information reaches the cerebral cortex. Information received from the diverse brain regions is passed on to the cortex through the thalamus. Anatomically, thalamus is the largest, most internal structure of the diencephalon consisting of dual lobe masses of gray matter. It is located at the rostral end of the midbrain on each side of the third ventricle. Each lobe is about 4 centimeters. Motor nuclei of the thalamus receive signals from the striatum and cerebellum and project into the motor and premotor areas of the cerebral cortex. The thalamus play a major role in the regulation of consciousness, alertness, arousal, and attention and is thus considered part of the limbic system.

Thalamus segmentation has become more and more essential for a wide range of clinical and research applications. For example, thalamus changes in terms of volume and intensity are involved in a large number of diseases, such as schizophrenia, Parkinson's disease and multiple sclerosis, and so forth. Manual segmentation is very labor intensive and the result is not reproducible. On the other hand, discrete methods such as thresholding or region growing are not reliable because of the low contrast and discontinuous edges in the MRI images of thalamus.

In this paper, we present a new semiautomatic framework for thalamus segmentation based on the concept of geometric surface flow. Unlike previous methods with edge information only, we apply a nonparametric kernel-based method that can simultaneously update the interior region statistics along with the boundary shape evolution. By integrating both boundary and region information, the new method is robust to image noise and inhomogeneity and will not leak from spurious edge gaps.

### 2. ACTIVE CONTOURS AND MEDICAL IMAGE SEGMENTATION

Segmenting structures from medical images and reconstructing a compact geometric representation of these structures is difficult due to the sheer size of the datasets and the complexity and variability of the anatomic shapes of interest. Furthermore, the shortcomings typical of sampled data, such as sampling artifacts, spatial aliasing, and noise, may cause the boundaries of structures to be indistinct and disconnected. The challenge is to extract boundary elements belonging to the same structure and integrate these elements into a coherent and consistent model of the structure. Among various segmentation techniques, active contours/deformable models have been very successful since their invention in late 80's. The mathematical foundation of deformable models stems from the confluence of geometry, physics, and approximation theory. Terzopoulos pioneered the theory of continuous (multidimensional) deformable models based on Lagrangian dynamics [1] and formulated deformation energies for generalized splines with controlled continuity [1]. Kass et al. [2] introduced the active contour model or "snake," a deformable model which is essentially a 2D spline that minimizes an internal deformation energy subject to external forces derived from images. Later, a voluminous literature on deformable models appeared in computer vision, and especially in the field of medical image analysis [3–9]. For a collection of seminal works, see [10, 11].

### 3. THALAMUS SEGMENTATION BY GEOMETRIC SURFACE FLOW

### 3.1. Geometric surface flow

Our new thalamus segmentation framework is based on the concept of geometric surface flow. The general formulation of the geometric surface flow is the following initial-value dynamical system of nonlinear PDEs:

$$\frac{\partial \vec{s}(\vec{p})}{\partial t} = F(t, k, k', f, \dots)\vec{n}(\vec{p}, t), \qquad \vec{s}(\vec{p}, 0) = \vec{s}_0(\vec{p}), \tag{1}$$

where $F$ is the speed function, $t$ is the time variable, $k$ and $k'$ are the surface curvature and its derivative at the point $\vec{p}$, $\vec{s}_0(\vec{p})$ is the initial surface, $\vec{n}$ is the surface normal vector. Equation (1) can be either directly provided by the user, or more generally, obtained as a gradient descent flow of the Euler-Lagrange equation of certain energy functionals by the calculus of variations.

In general, there are two approaches to numerically simulate PDEs such as (1): explicit Lagrangian approach or implicit level-set approach. Implicit level-set methods [3, 6] are becoming popular recently mainly because of their ease in handling topology changes, however since all the computations are conducted in a higher dimension, the computational cost tends to be quite expensive. In this paper, we take the explicit Lagrangian approach to simulate the surface flow. In particular, we apply the new framework we recently proposed [12, 13]. In our new framework, the geometry and topology of the model are always explicitly represented throughout the simulation process. To ensure the regularity of the model and the stability of the numerical integration process, powerful Laplacian tangential smoothing, along with commonly used mesh optimization techniques, are employed throughout the geometric deformation and topological variation process. In addition, a novel particle-based collision detection scheme is conducted to automatically handle topology changes during the deformation. The new framework for surface flow simulation is fast, simple, and accurate. More importantly, it allows the user to directly interact with the model during the deformation. For more details of our new framework, please refer to [12, 13].

### 3.2. Thalamus segmentation

When applying the above geometric surface flow for thalamus segmentation, the speed function $F$ in (1) is explicitly formulated as a linear combination of the following two terms:

$$F = a_1 F_{\text{reg}} + F_{\text{data}}, \tag{2}$$

here $F_{\text{reg}}$ is the regularity term to maintain the smoothness of the model. $F_{\text{reg}}$ is usually a function of the curvature (e.g., mean curvature, Gaussian curvature). In this paper, we define $F_{\text{reg}}$ as the difference between the mean curvature $H_{\text{curr}}$ at the current position and the average mean curvature $H_{\text{avg}}$ of the whole model:

$$F_{\text{reg}} = H_{\text{curr}} - H_{\text{avg}}. \tag{3}$$

Comparing with other commonly used regularity terms such as mean curvature or Gaussian curvature, (3) works much better in our experiment. This is probably because of the volume preserving property of (3), which can be considered as a shape prior term for the thalamus's oval shape. A more detailed explanation can be found in [14], where a similar term is used.

$F_{\text{data}}$ is the term that interacts the model with the image data, and is often defined as a function of the edge information of the image data. However, since the thalamus has low contrast, using boundary information alone is not very reliable. It is more preferable to combine the edge information along with the region information as suggested by Huang et al. [15]. Hence, in this paper, we propose to employ both the edge information as well as the region information. In particular, we define $F_{\text{data}}$ as a linear combination of the following two terms:

$$F_{\text{data}} = a_2 F_{\text{edge}} + a_3 F_{\text{region}}, \tag{4}$$

$F_{\text{edge}}$ is the speed function corresponding to the edge information in the image data that will attract the contour moving towards the edges of the image data and is defined as

$$F_{\text{edge}} = -\Delta(G_\sigma * I(s)), \tag{5}$$

where $\Delta$ is the Laplacian operator, $I$ is the image intensity function, and $G_\sigma * I$ is the smoothed intensity function by convoluting with a Gaussian filter with variance $\sigma$. Variance $\sigma$ can be assigned according to the image resolution of the MRI dataset. In our experiment, $\sigma$ is set as 1.0.

$F_{\text{region}}$ is the speed function corresponding to the region information, and is defined as

$$F_{\text{region}} = \frac{1}{1 + |\nabla(B(s))|^2}, \tag{6}$$

where $\nabla$ is the gradient operator, $B(s)$ is the binary image created by the interior probability estimation of the current model, which will be explained in more detail in the following section (Sections 3.3.2 and 3.3.3). In a nutshell, $F_{\text{region}}$ will expand the contour outwards in regions that are considered compatible with the current segmented volume's statistics.

Substituting (3), (4), (5), and (6) into (2), we obtained the following speed function for thalamus segmentation:

$$F = a_1(H_{\text{curr}} - H_{\text{avg}}) - a_2\Delta(G_\sigma * I(s)) + a_3\frac{1}{1 + |\nabla(B(s))|^2}. \tag{7}$$

Here, $a_1$, $a_2$, $a_3$ are the corresponding weighting coefficients. In our experiments, $a_1$, $a_2$, $a_3$ are set as 0.1, 1, 1, respectively.

### 3.3. Algorithm pipeline

There are four main steps in our thalamus segmentation framework: (1) seed initialization; (2) interior statistics estimation; (3) binary image creation; (4) model evolution.

#### 3.3.1. Seed initialization

First, the user interactively selects a pixel/voxel inside the region of interest in the image data. Considering the thalamus has an oval shape, a circular contour centered at the pixel/voxel is then automatically created and serves as the initial seed model.

#### 3.3.2. Interior statistics estimation

Then, the intensity probability density function of the interior regions enclosed by the seed contour is estimated. Specifically, we approximate the distribution by a Parzen-window-function-based nonparametric method [16] because it is differentiable, more generic, and can represent complex multimodal intensity distributions. We choose the Gaussian kernel as the Parzen window function. Suppose the model $M$ is placed on an image $I$, the volume of the image region bounded by the current model $M$ is $V$, then the probability of a pixel's (voxel's) intensity value $i$ being consistent with the model interior intensity can be derived as

$$P(i \mid M) = \frac{1}{V}\iiint \frac{1}{\sqrt{2\pi}\sigma}e^{-(i-I(y))^2/2\sigma^2}, \tag{8}$$

where $\sigma$ is a constant that specifies the width of the Gaussian kernel and is set as 1.0 in our experiment. Since (8) is a simple integral, it can be calculated very efficiently as an incremental update, that is, only newly added voxels are calculated to update the integral value at each time step.

#### 3.3.3. Binary image creation

Next, based on the interior probability density distribution of model $M$ obtained in the previous step, the image intensity probability map $P_I$ of every pixel's (voxel's) intensity is obtained. Then a small threshold (e.g., the mean probability over the entire image domain) is applied on $P_I$ to produce a binary image $B(s)$, in which pixels/voxels with probability higher than the threshold have value 1, zero otherwise.

#### 3.3.4. Model evolution

Finally, the model will start to evolve according to (1) and (7). More specifically, the surface evolution process is numerically approximated using a simple, explicit iterative equation:

$$\vec{S}(\vec{p}, t + \Delta t) = \vec{S}(\vec{p}, t) + F(\vec{p}, t)\vec{N}(\vec{p}, t)\Delta t. \tag{9}$$

When advancing the model, we must enforce a constraint on the size of the time step $\Delta t$. In particular, the time step $\Delta t$ must satisfy the following stability condition: the velocity of change must be strictly restrained by the minimum detail in the system. In our system, this condition is

$$\Delta t \le \frac{m_e}{M_F}, \tag{10}$$

where $m_e$ is the unit grid cell length of the image data and $M_F$ is the maximum magnitude of the speed $F$ obtained by (7). Before each deformation step, we will calculate the velocity $F$ at each vertex point and determine the maximum magnitude of the velocity $M_F$. A proper time step can then be estimated from (10). At each deformation cycle, the model will loop from step 2 to step 4 until an equilibrium state is achieved, then the deformation stops and the geometry of the thalamus is extracted from the MRI image.

### 3.4. Experimental results

Some of the experimental results for 2D and 3D thalamus segmentations are shown in Figure 1 to Figure 4. In particular, an example of our 2D thalamus segmentation result is shown in Figures 1 and 2. Each of the two figures shows five snapshots of the model evolution process including the initial seed (a) and the final shape (e). Figure 1 shows the model superimposed with the original image, while Figure 2 shows the same five snapshots superimposed with the binary image created by the interior statistics estimation (Sections 3.3.2 and 3.3.3). The 3D thalamus segmentation result is shown in Figures 3 and 4. Figure 3 shows the four snapshots during the model evolution process. The four different views of the final 3D shape of the thalamus are shown in Figure 4.

### 4. CONCLUSION

We proposed a semiautomatic thalamus segmentation method based on an explicit simulation of geometric surface flow. To overcome the low contrast of the thalamus, we employ a nonparametric kernel-based statistics estimation that can incorporate both the boundary and region information in the

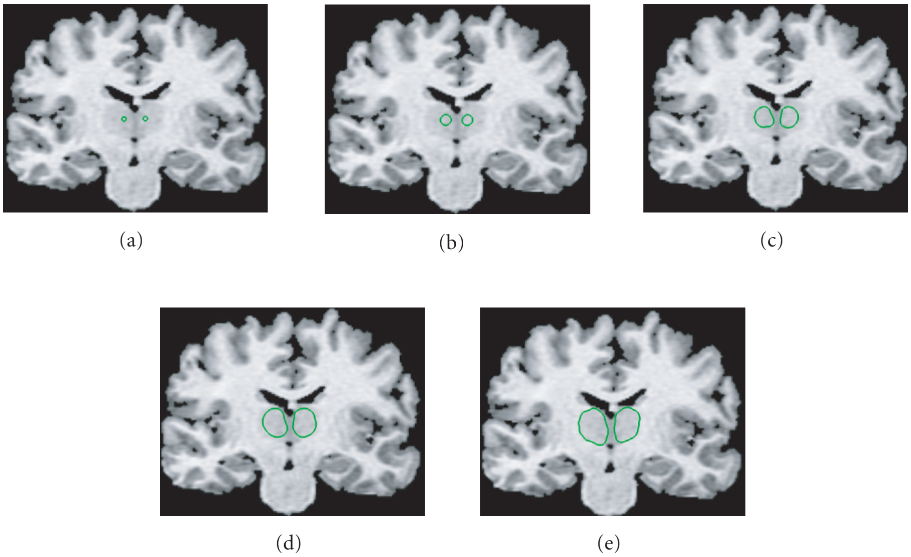

FIGURE 1: The model evolution process of the 2D thalamus segmentation superimposed with original image. (a) Initial seed; (b)–(d) three intermediate stages; and (e) final extracted shape.

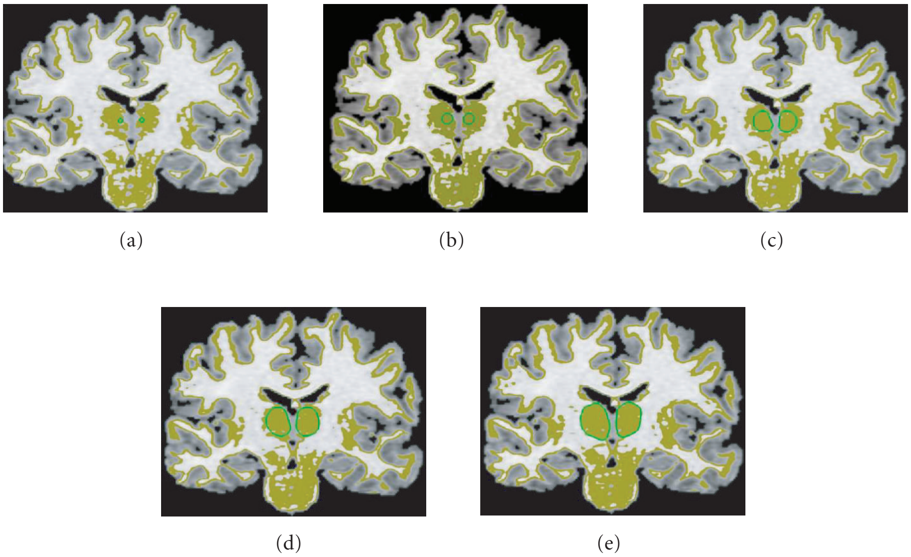

FIGURE 2: The same five snapshots of the model evolution process of the 2D thalamus segmentation superimposed with the binary mask created by interior statistics estimation.

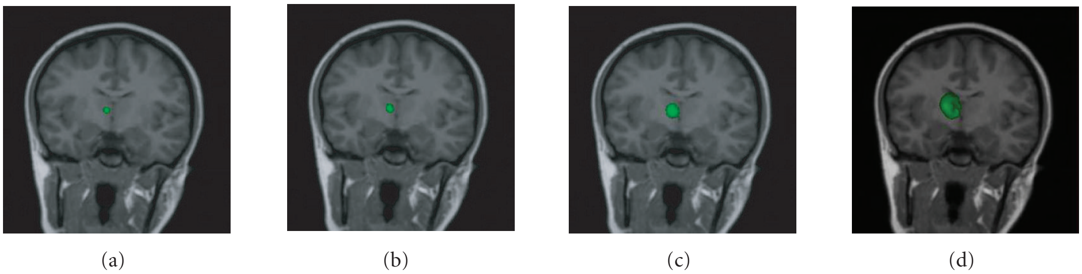

FIGURE 3: The model evolution process of the 3D thalamus segmentation superimposed with original image. (a) Initial seed; (b)-(c) two intermediate stages; and (d) final extracted shape.

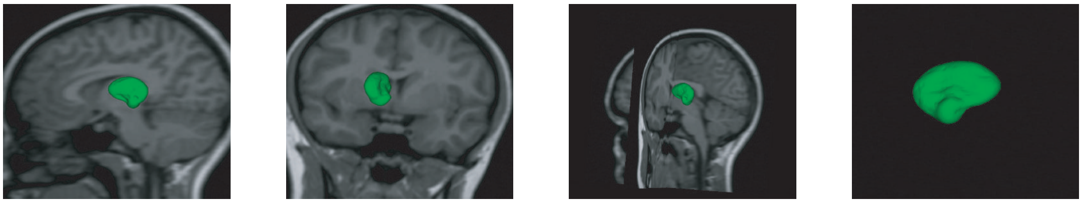

(a) (b) (c) (d)

FIGURE 4: (a)–(c) Three different views of the final shape of the 3D thalamus; and (d) a close-up view of the thalamus.

model evolution process, so that the model is very robust to image noise and small gaps. In the future we would also like to apply our method for the more challenging task of thalamus nuclei segmentation from the newly available diffusion tensor images.

### ACKNOWLEDGMENT

The brain MRI dataset used in this paper is kindly provided by Dr. Otto Muzik from the PET Imaging Center, School of Medicine, Wayne State University, Detroit, Michigan.

### REFERENCES

[1] D. Terzopoulos, "Regularization of inverse visual problems involving discontinuities," *IEEE Transactions on Pattern Analysis and Machine Intelligence*, vol. 8, no. 4, pp. 413–424, 1986.

[2] M. Kass, A. Witkin, and D. Terzopoulos, "Snakes: active contour models," *International Journal of Computer Vision*, vol. 1, no. 4, pp. 321–331, 1988.

[3] V. Caselles, R. Kimmel, G. Sapiro, and C. Sbert, "Minimal surfaces based object segmentation," *IEEE Transactions on Pattern Analysis and Machine Intelligence*, vol. 19, no. 4, pp. 394–398, 1997.

[4] H. Delingette and J. Montagnat, "Shape and topology constraints on parametric active contours," *Computer Vision and Image Understanding*, vol. 83, no. 2, pp. 140–171, 2001.

[5] J.-O. Lachaud and A. Montanvert, "Deformable meshes with automated topology changes for coarse-to-fine three-dimensional surface extraction," *Medical Image Analysis*, vol. 3, no. 2, pp. 187–207, 1999.

[6] R. Malladi, J. A. Sethian, and B. C. Vemuri, "Shape modeling with front propagation: a level set approach," *IEEE Transactions on Pattern Analysis and Machine Intelligence*, vol. 17, no. 2, pp. 158–175, 1995.

[7] T. McInerney and D. Terzopoulos, "Topology adaptive deformable surfaces for medical image volume segmentation," *IEEE Transactions on Medical Imaging*, vol. 18, no. 10, pp. 840–850, 1999.

[8] D. Metaxas and D. Terzopoulos, "Shape and nonrigid motion estimation through physics-based synthesis," *IEEE Transactions on Pattern Analysis and Machine Intelligence*, vol. 15, no. 6, pp. 580–591, 1993.

[9] J. V. Miller, D. E. Breen, W. E. Lorensen, R. M. O'Bara, and M. J. Wozny, "Geometrically deformed models: a method for extracting closed geometric models form volume data," in *Proceedings of 18th Annual Conference on Computer Graphics and Interactive Techniques (SIGGRAPH '91)*, pp. 217–226, Las Vegas, Nev, USA, July–August 1991.

[10] T. McInerney and D. Terzopoulos, "Deformable models in medical image analysis: a survey," *Medical Image Analysis*, vol. 1, no. 2, pp. 91–108, 1996.

[11] A. Singh, D. Goldgof, and D. Terzopoulos, Eds., *Deformable Models in Medical Image Analysis*, IEEE Computer Society Press, Los Alamitos, Calif, USA, 1998.

[12] Y. Duan, J. Hua, and H. Qin, "HapticFlow: PDE-based mesh editing with haptics," *Computer Animation & Virtual Worlds*, vol. 15, no. 3-4, pp. 193–200, 2004.

[13] Y. Duan, J. Hua, and H. Qin, "Interactive shape modeling using lagrangian surface flow," *The Visual Computer*, vol. 21, no. 5, pp. 279–288, 2005.

[14] H.-K. Zhao, B. Merriman, S. Osher, and L. Wang, "Capturing the behavior of bubbles and drops using the variational level set approach," *Journal of Computational Physics*, vol. 143, no. 2, pp. 495–518, 1998.

[15] X. Huang, D. Metaxas, and T. Chen, "MetaMorphs: Deformable shape and texture models," in *Proceedings of IEEE Conference on Computer Vision and Pattern Recognition (CVPR '04)*, vol. 1, pp. 496–503, Washington, DC, USA, June–July 2004.

[16] C. M. Bishop, *Neural Networks for Pattern Recognition*, Oxford University Press, Oxford, UK, 1995.

**Greg Heckenberg** received his B.S. degree in computer science from University of Missouri-Columbia in May 2004. He is currently pursuing his M.S. degree in computer science. His areas of interest include image segmentation and understanding, volumetric graphics, machine learning, physically based simulations, and deformable models.

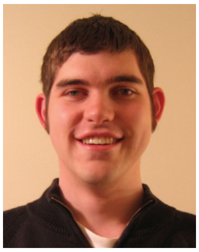

**Yongjian Xi** received B.S. and M.E. degrees in electrical engineering from Tsinghua University, China, in 1994 and 1997, received M.S. degree in computer science from Polytechnic University, New York, USA, in 2001. He is presently pursuing a Ph.D. degree in computer science in the University of Missouri-Columbia. His research interests includes 3D model reconstrution and volumetric data visualization.

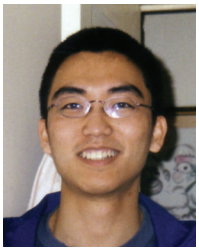

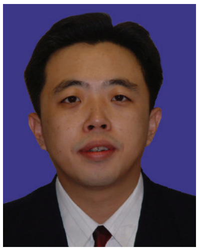

**Ye Duan** is an Assistant Professor of computer science at University of Missouri-Columbia. He received his Ph.D. degree in computer science from the State University of New York at Stony Brook (2003). He received his M.S. degree in computer science from the State University of New York at Stony Brook in 1998. In 1996, he received his M.S. degree in mathematics from Utah State University. He received his B.A. degree in mathematics from Peking University in 1991. His research interests include biomedical imaging, computer graphics, scientific visualization, computer vision, geometric and physics-based modeling, virtual reality and human-computer Interaction, and computer animation and simulation.

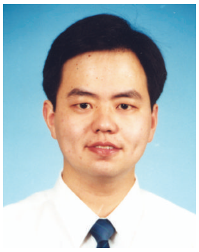

**Jing Hua** is an Assistant Professor in computer science at the Wayne State University and he is the Director of Graphics and Imaging Lab. He received his Ph.D. degree in computer science from the State University of New York at Stony Brook in 2004. His research interests include biomedical image analysis, computational geometry, physics-based modeling, scientific visualization, human-computer interaction, and computer vision.

